# Supplementary material for: Biophysical mapping of TREM2-ligand interactions reveals shared surfaces for engagement of multiple Alzheimer’s disease ligands
Source: Mol Neurodegener. 2025 Jan 9;20:3. doi: 10.1186/s13024-024-00795-9 (PMC11721465; doi:10.1186/s13024-024-00795-9)
Supplement: Supplementary file 1 — Supplementary Material 1 [file 13024_2024_795_MOESM1_ESM.docx]

**Supplemental data.**

**Table S1.** Binding sites on TREM2 basic site and CDR2 regions for IL-34 helices predicted by hydropathy mapping to have at least 75% percent match and a degree of complementary hydropathy (DCH) of at least 0.5.

| Region (Residues) | | Forward | | | | Reverse | | | |
| --- | --- | --- | --- | --- | --- | --- | --- | --- | --- |
| IL-34 | TREM2 | Match | DCH | Start | End | Match | DCH | Start | End |
| Helix 2  (67-82) | Basic site and CDR2  (62-78) | 75.00% | 0.533 | 60 | 75 | 75.00% | 0.545 | 60 | 75 |
|  | CDR2 and Basic site  (69-78) |  |  |  |  | 75.00% | 0.54 | 71 | 86 |
|  | Basic site  (112-114) |  |  |  |  | 75.00% | 0.616 | 112 | 127 |
| Helix 5  (139-151) | Basic site  (47-50) | 76.92% | 0.503 | 49 | 61 |  |  |  |  |
|  | Basic site  (62-68) |  |  |  |  | 76.92% | 0.539 | 53 | 65 |
|  | CDR2 and Basic site  (69-78) | 76.92% | 0.538 | 70 | 82 |  |  |  |  |
|  | Basic site  (112-114) |  |  |  |  | 84.62% | 0.573 | 114 | 126 |
|  | Other |  |  |  |  | 84.62% | 0.568 | 117 | 129 |
| Helix6  (160-181) | Basic site and CDR2  (47-75) |  |  |  |  | 77.27% | 0.521 | 49 | 70 |
|  | Basic site  (112-114) | 77.27% | 0.552 | 100 | 121 |  |  |  |  |

**Table S2.** Potential binding regions on IL-34 for TREM2 predicted by hydropathy mapping to have at least 75% percent match and a degree of complementary (DCH) hydropathy of at least 0.5. Note: Rows with text colored in red represent the top predicted binding site(s) between a pair of regions.

| Region (Residues) | | Forward | | | | Reverse | | | |
| --- | --- | --- | --- | --- | --- | --- | --- | --- | --- |
| TREM2 | IL-34 | Match | DCH | Start | End | Match | DCH | Start | End |
| Basic 1  (47-50) | Negative Electrostatic Surface Potential | 75.00% | 0.664 | 40 | 43 | 75.00% | 0.511 | 37 | 40 |
|  |  |  |  |  |  | 75.00% | 0.528 | 64 | 67 |
|  |  |  |  |  |  | 75.00% | 0.550 | 83 | 86 |
|  |  | 75.00% | 0.519 | 92 | 95 | 75.00% | 0.608 | 90 | 93 |
|  |  | 100.00% | 0.658 | 96 | 99 | 100.00% | 0.661 | 93 | 96 |
|  |  |  |  |  |  | 75.00% | 0.531 | 97 | 100 |
|  |  | 75.00% | 0.542 | 105 | 108 | 75.00% | 0.542 | 105 | 108 |
|  |  | 75.00% | 0.606 | 109 | 112 | 75.00% | 0.675 | 106 | 109 |
|  |  | 75.00% | 0.531 | 119 | 122 | 75.00% | 0.531 | 119 | 122 |
|  |  |  |  |  |  | 75.00% | 0.608 | 122 | 125 |
|  |  |  |  |  |  | 75.00% | 0.586 | 123 | 126 |
|  |  | 100.00% | 0.733 | 126 | 129 | 75.00% | 0.531 | 127 | 130 |
|  |  | 75.00% | 0.608 | 133 | 136 | 75.00% | 0.617 | 136 | 139 |
|  |  | 75.00% | 0.542 | 135 | 138 | 75.00% | 0.617 | 142 | 145 |
|  |  |  |  |  |  | 75.00% | 0.600 | 143 | 146 |
|  |  | 100.00% | 0.658 | 145 | 148 | 100.00% | 0.647 | 146 | 149 |
|  |  | 100.00% | 0.667 | 148 | 151 | 75.00% | 0.517 | 149 | 152 |
|  |  | 100.00% | 0.681 | 171 | 174 | 100.00% | 0.669 | 172 | 175 |
|  |  | 100.00% | 0.625 | 174 | 177 | 100.00% | 0.539 | 177 | 180 |
|  |  | 75.00% | 0.603 | 179 | 182 | 75.00% | 0.531 | 183 | 186 |
|  |  | 75.00% | 0.603 | 185 | 188 | 75.00% | 0.547 | 191 | 194 |
|  | Positive Electrostatic Surface Potential | 75.00% | 0.519 | 57 | 60 | 75.00% | 0.528 | 51 | 54 |
|  |  | 100.00% | 0.753 | 71 | 74 | 75.00% | 0.561 | 69 | 72 |
|  |  | 100.00% | 0.697 | 74 | 77 | 100.00% | 0.686 | 72 | 75 |
|  |  | 75.00% | 0.636 | 77 | 80 | 75.00% | 0.553 | 75 | 78 |
|  |  | 75.00% | 0.531 | 155 | 158 | 100.00% | 0.744 | 156 | 159 |
|  |  | 75.00% | 0.650 | 158 | 161 | 75.00% | 0.567 | 161 | 164 |
|  |  | 75.00% | 0.664 | 164 | 167 | 75.00% | 0.600 | 166 | 169 |
|  |  | 100.00% | 0.697 | 168 | 171 | 100.00% | 0.681 | 169 | 172 |
|  | Other | 75.00% | 0.519 | 23 | 26 | 75.00% | 0.539 | 24 | 27 |
|  |  | 75.00% | 0.542 | 209 | 212 | 75.00% | 0.525 | 205 | 208 |
|  |  |  |  |  |  | 75.00% | 0.622 | 230 | 233 |
|  |  | 75.00% | 0.556 | 232 | 235 | 75.00% | 0.503 | 233 | 236 |
|  |  |  |  |  |  | 75.00% | 0.578 | 238 | 241 |
| Basic 2  (62-68) | Negative Electrostatic Surface Potential | 85.71% | 0.667 | 90 | 96 |  |  |  |  |
|  |  | 85.71% | 0.644 | 105 | 111 |  |  |  |  |
|  |  | 85.71% | 0.594 | 122 | 128 |  |  |  |  |
|  |  | 85.71% | 0.644 | 142 | 148 |  |  |  |  |
|  | Positive Electrostatic Surface Potential | 85.71% | 0.684 | 159 | 165 | 85.71% | 0.678 | 74 | 80 |
|  |  | 85.71% | 0.627 | 165 | 171 | 85.71% | 0.586 | 162 | 168 |
| Basic 3  (76-78) | Negative Electrostatic Surface Potential | 100.00% | 0.819 | 95 | 97 | 100.00% | 0.819 | 95 | 97 |
|  |  | 100.00% | 0.804 | 108 | 110 | 100.00% | 0.804 | 108 | 110 |
|  |  | 100.00% | 0.789 | 125 | 127 | 100.00% | 0.789 | 125 | 127 |
|  | Positive Electrostatic Surface Potential | 100.00% | 0.715 | 163 | 165 | 100.00% | 0.715 | 163 | 165 |
| Basic 4  (112-114) | Negative Electrostatic Surface Potential | 100.00% | 0.641 | 64 | 66 | 100.00% | 0.641 | 64 | 66 |
|  |  | 100.00% | 0.641 | 83 | 85 | 100.00% | 0.641 | 83 | 85 |
|  |  | 100.00% | 0.619 | 93 | 95 | 100.00% | 0.619 | 93 | 95 |
|  |  | 100.00% | 0.615 | 97 | 99 | 100.00% | 0.615 | 97 | 99 |
|  |  | 100.00% | 0.526 | 99 | 101 | 100.00% | 0.526 | 99 | 101 |
|  |  | 100.00% | 0.715 | 127 | 129 | 100.00% | 0.715 | 127 | 129 |
|  |  | 100.00% | 0.730 | 136 | 138 | 100.00% | 0.730 | 136 | 138 |
|  |  | 100.00% | 0.600 | 146 | 148 | 100.00% | 0.600 | 146 | 148 |
|  |  | 100.00% | 0.626 | 149 | 151 | 100.00% | 0.626 | 149 | 151 |
|  |  | 100.00% | 0.630 | 172 | 174 | 100.00% | 0.630 | 172 | 174 |
|  |  | 100.00% | 0.570 | 175 | 177 | 100.00% | 0.570 | 175 | 177 |
|  |  | 100.00% | 0.504 | 177 | 179 | 100.00% | 0.504 | 177 | 179 |
|  |  | 100.00% | 0.667 | 191 | 193 | 100.00% | 0.667 | 191 | 193 |
|  | Positive Electrostatic Surface Potential | 100.00% | 0.563 | 35 | 37 | 100.00% | 0.563 | 35 | 37 |
|  |  | 100.00% | 0.619 | 58 | 60 | 100.00% | 0.619 | 58 | 60 |
|  |  | 100.00% | 0.726 | 72 | 74 | 100.00% | 0.726 | 72 | 74 |
|  |  | 100.00% | 0.641 | 75 | 77 | 100.00% | 0.641 | 75 | 77 |
|  |  | 100.00% | 0.715 | 156 | 158 | 100.00% | 0.715 | 156 | 158 |
|  |  | 100.00% | 0.715 | 169 | 171 | 100.00% | 0.715 | 169 | 171 |
|  | Other | 100.00% | 0.630 | 24 | 26 | 100.00% | 0.630 | 24 | 26 |
|  |  | 100.00% | 0.678 | 233 | 235 | 100.00% | 0.678 | 233 | 235 |
| CDR1  (39-46) | Negative Electrostatic Surface Potential | 75.00% | 0.586 | 90 | 97 | 75.00% | 0.542 | 93 | 100 |
|  |  | 75.00% | 0.535 | 92 | 99 | 75.00% | 0.503 | 95 | 102 |
|  |  | 75.00% | 0.614 | 122 | 129 |  |  |  |  |
|  |  | 75.00% | 0.567 | 142 | 149 | 75.00% | 0.578 | 142 | 149 |
|  |  | 75.00% | 0.519 | 145 | 152 | 75.00% | 0.522 | 145 | 152 |
|  |  | 75.00% | 0.521 | 171 | 178 | 75.00% | 0.521 | 171 | 178 |
|  | Positive and Negative Electrostatic Surface Potential | 87.50% | 0.615 | 168 | 175 | 87.50% | 0.615 | 168 | 175 |
|  | Positive Electrostatic Surface Potential | 75.00% | 0.585 | 71 | 78 | 75.00% | 0.585 | 71 | 78 |
|  |  | 75.00% | 0.513 | 158 | 165 |  |  |  |  |
|  |  | 75.00% | 0.554 | 164 | 171 |  |  |  |  |
|  |  | 75.00% | 0.589 | 165 | 172 | 75.00% | 0.600 | 165 | 172 |
| CDR2  (69-75) | Negative Electrostatic Surface Potential | 85.71% | 0.632 | 86 | 92 | 100.00% | 0.667 | 88 | 94 |
|  |  | 85.71% | 0.541 | 89 | 95 | 85.71% | 0.565 | 103 | 109 |
|  |  | 85.71% | 0.565 | 115 | 121 | 85.71% | 0.538 | 114 | 120 |
|  |  | 100.00% | 0.638 | 118 | 124 | 100.00% | 0.689 | 117 | 123 |
|  |  | 85.71% | 0.514 | 132 | 138 | 85.71% | 0.557 | 120 | 126 |
|  |  | 85.71% | 0.560 | 141 | 147 | 100.00% | 0.624 | 131 | 137 |
|  |  | 85.71% | 0.503 | 176 | 182 | 85.71% | 0.541 | 140 | 146 |
|  | Positive and Negative Electrostatic Surface Potential | 85.71% | 0.602 | 79 | 85 |  |  |  |  |
|  | Positive Electrostatic Surface Potential | 85.71% | 0.606 | 28 | 34 | 85.71% | 0.562 | 27 | 33 |
|  |  |  |  |  |  | 85.71% | 0.571 | 30 | 36 |
|  |  | 85.71% | 0.641 | 44 | 50 | 85.71% | 0.622 | 43 | 49 |
|  |  | 85.71% | 0.597 | 47 | 53 | 85.71% | 0.635 | 46 | 52 |
|  |  | 100.00% | 0.663 | 50 | 56 | 100.00% | 0.625 | 49 | 55 |
|  |  | 85.71% | 0.632 | 73 | 79 | 85.71% | 0.605 | 75 | 81 |
|  |  | 100.00% | 0.705 | 76 | 82 | 100.00% | 0.667 | 78 | 84 |
|  |  | 85.71% | 0.594 | 155 | 161 | 85.71% | 0.594 | 154 | 160 |
|  | Other | 100.00% | 0.565 | 25 | 31 | 85.71% | 0.511 | 197 | 203 |
|  |  | 85.71% | 0.541 | 192 | 198 | 85.71% | 0.535 | 200 | 206 |
|  |  | 85.71% | 0.540 | 201 | 207 | 85.71% | 0.508 | 210 | 216 |
|  |  | 85.71% | 0.525 | 211 | 217 | 85.71% | 0.522 | 225 | 231 |
|  |  | 85.71% | 0.598 | 229 | 235 | 100.00% | 0.633 | 228 | 234 |
| CDR3  (88-91) | Negative Electrostatic Surface Potential | 100.00% | 0.5 | 93 | 96 | 75.00% | 0.514 | 40 | 43 |
|  |  | 75.00% | 0.553 | 105 | 108 | 75.00% | 0.514 | 82 | 85 |
|  |  | 75.00% | 0.525 | 106 | 109 | 75.00% | 0.553 | 105 | 108 |
|  |  | 75.00% | 0.542 | 119 | 122 | 75.00% | 0.542 | 119 | 122 |
|  |  | 75.00% | 0.542 | 127 | 130 | 100.00% | 0.572 | 126 | 129 |
|  |  |  |  |  |  | 75.00% | 0.553 | 135 | 138 |
|  |  |  |  |  |  | 100.00% | 0.506 | 148 | 151 |
|  |  | 100.00% | 0.508 | 172 | 175 | 100.00% | 0.519 | 171 | 174 |
|  |  |  |  |  |  | 75.00% | 0.506 | 190 | 193 |
|  | Positive Electrostatic Surface Potential | 100.00% | 0.525 | 72 | 75 | 100.00% | 0.592 | 71 | 74 |
|  |  |  |  |  |  | 100.00% | 0.536 | 74 | 77 |
|  |  | 75.00% | 0.503 | 80 | 83 | 75.00% | 0.503 | 80 | 83 |
|  |  |  |  |  |  | 75.00% | 0.542 | 155 | 158 |
|  |  | 100.00% | 0.583 | 156 | 159 | 75.00% | 0.5 | 158 | 161 |
|  |  |  |  |  |  | 75.00% | 0.514 | 164 | 167 |
|  |  | 100.00% | 0.519 | 169 | 172 | 100.00% | 0.536 | 168 | 171 |
|  | Other | 75.00% | 0.528 | 230 | 233 | 75.00% | 0.528 | 230 | 233 |
|  |  | 75.00% | 0.514 | 233 | 236 |  |  |  |  |

**Supplemental Figure 1.
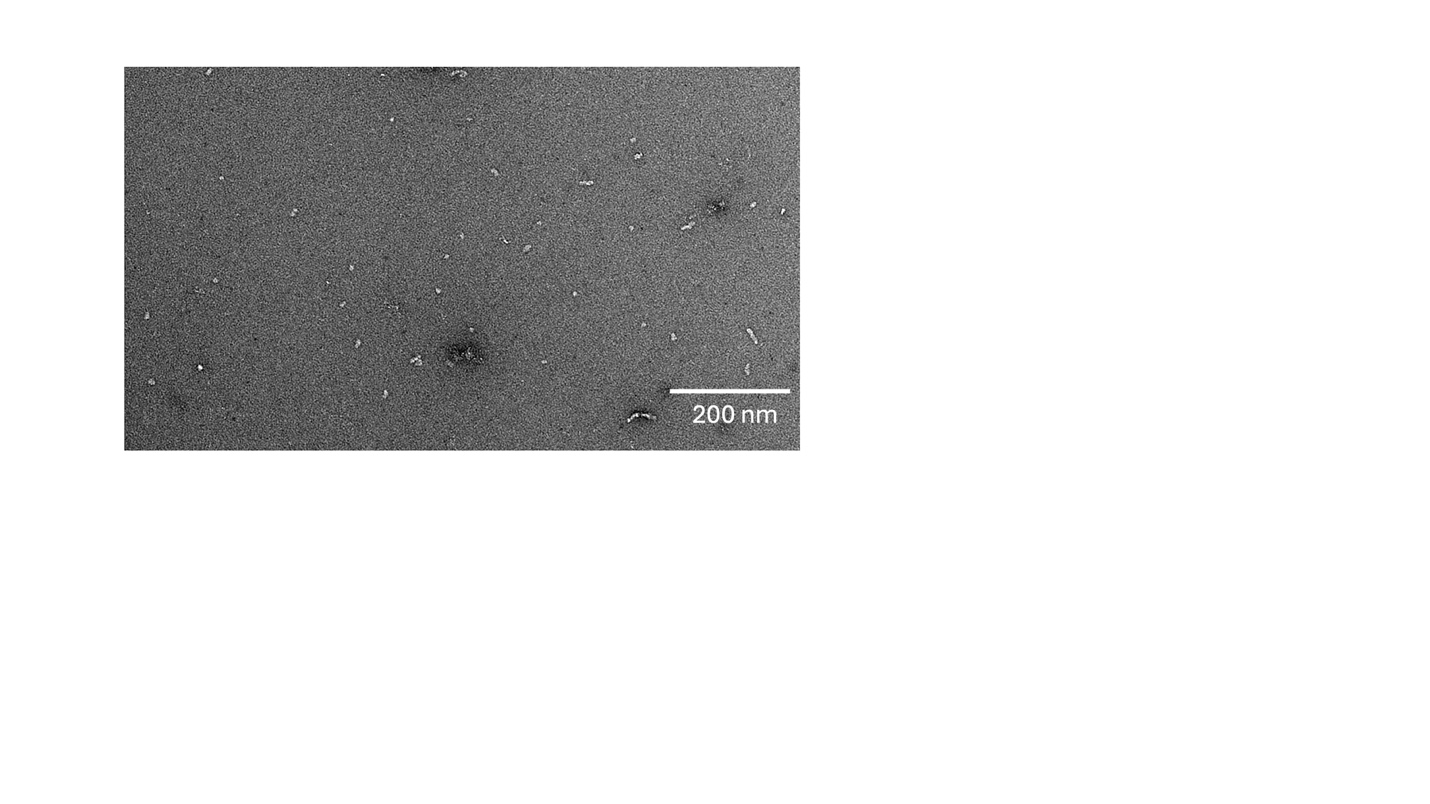
TEM of oAβ42 preparations.** TEM shows amorphous oligomers (small round and oval spots) and some short protofibrils (squiggles).

**
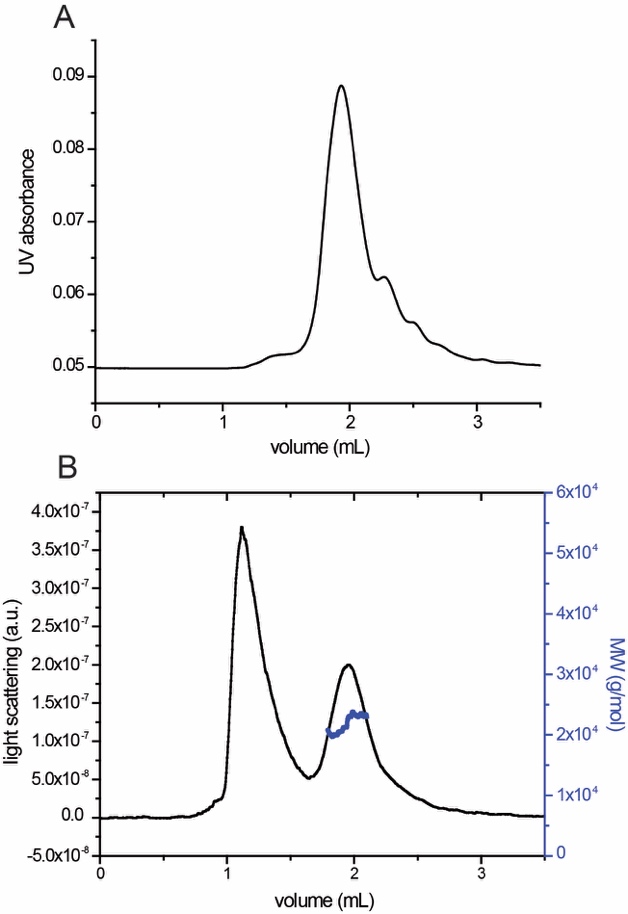
**

**Supplemental Figure 2. SEC-MALS analysis WT sTREM2 shows a monomer in solution**. SEC-MALS analysis showing the (A) absorbance and (B) light scattering of WT sTREM2. Mass estimate from Rayleigh ratio is shown in blue (MW_avg_ = 21.3 ± 0.5 kDa [MW_calc_ = 17.0 kDa]; Polydispersity = 1.002 ± 0.001; n = 3).

**Supplemental Figure 3.
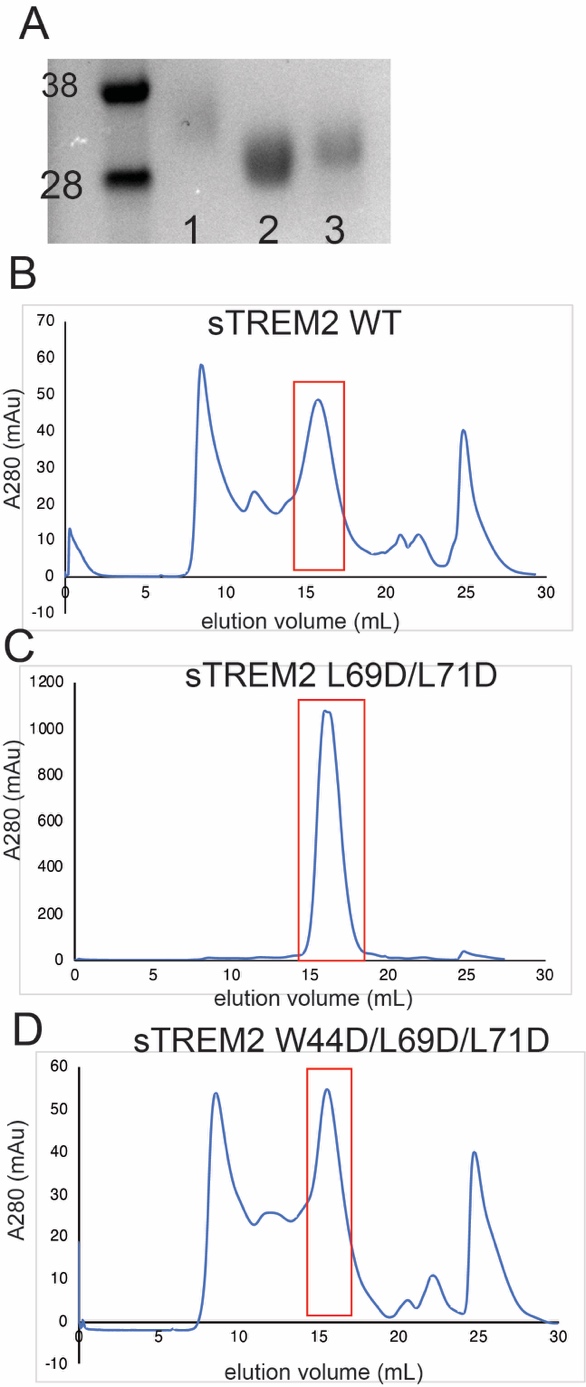
sTREM2 gel and s200 SEC chromatograms A)** protein gel with sTREM2 WT in lane 1, sTREM2 L69D/L71D in lane 2, and sTREM2 W44D/L69D/L71D in lane 3 **B-D)** s200 SEC chromatograms for sTREM2 **B)** WT, **C)** sTREM2 L69D/L71D, **D)** sTREM2 W44D/L69D/L71D.


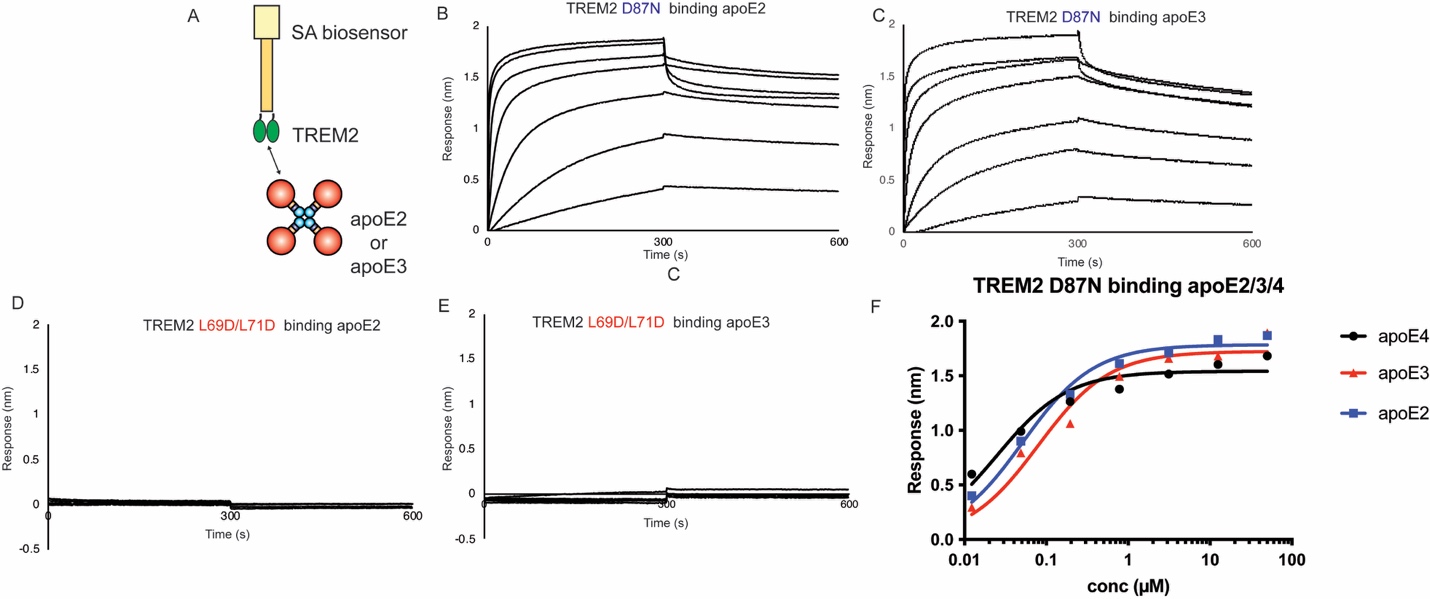


**Supplemental Figure 4**. **ApoE2, apoE3, and apoE4 bind TREM2 variants similarly.** Immobilized TREM2 WT and variants were probed for binding to apoE2 and apoE3 (0.012 - 50 μM). **(A)** Scheme of experiment. **B-E)** BLI sensorgrams for B) TREM2 D87N binding apoE2, **C)**TREM2 D87N binding apoE3, **D)** TREM2 L69D/L71D binding apoE2, and **E)** TREM2 L69D/L71D binding apoE3, Double-reference subtracted data shown in black. **F)** Steady state analysis and non-linear fits to derive K_D_ from data shown in **B&C**. The derived K_D_s are: TREM2 D87N/apoE2 = 52 nM; TREM2 D87N/apoE3 = 79 nM; TREM2 D87N/apoE4 = 25 nM.

**Supplemental Figure 5**. **ELISA.** Immobilized apoE4 was plated, washed, and probed for binding to TREM2 WT and variants. Replicates were carried out (n=6). Absorbance readings were plotted in Prism as mean +/- standard error of the mean and statistics were analyzed by One-way ANOVA. ***p < 0.05, *p < 0.01,*******p < 0.001.**


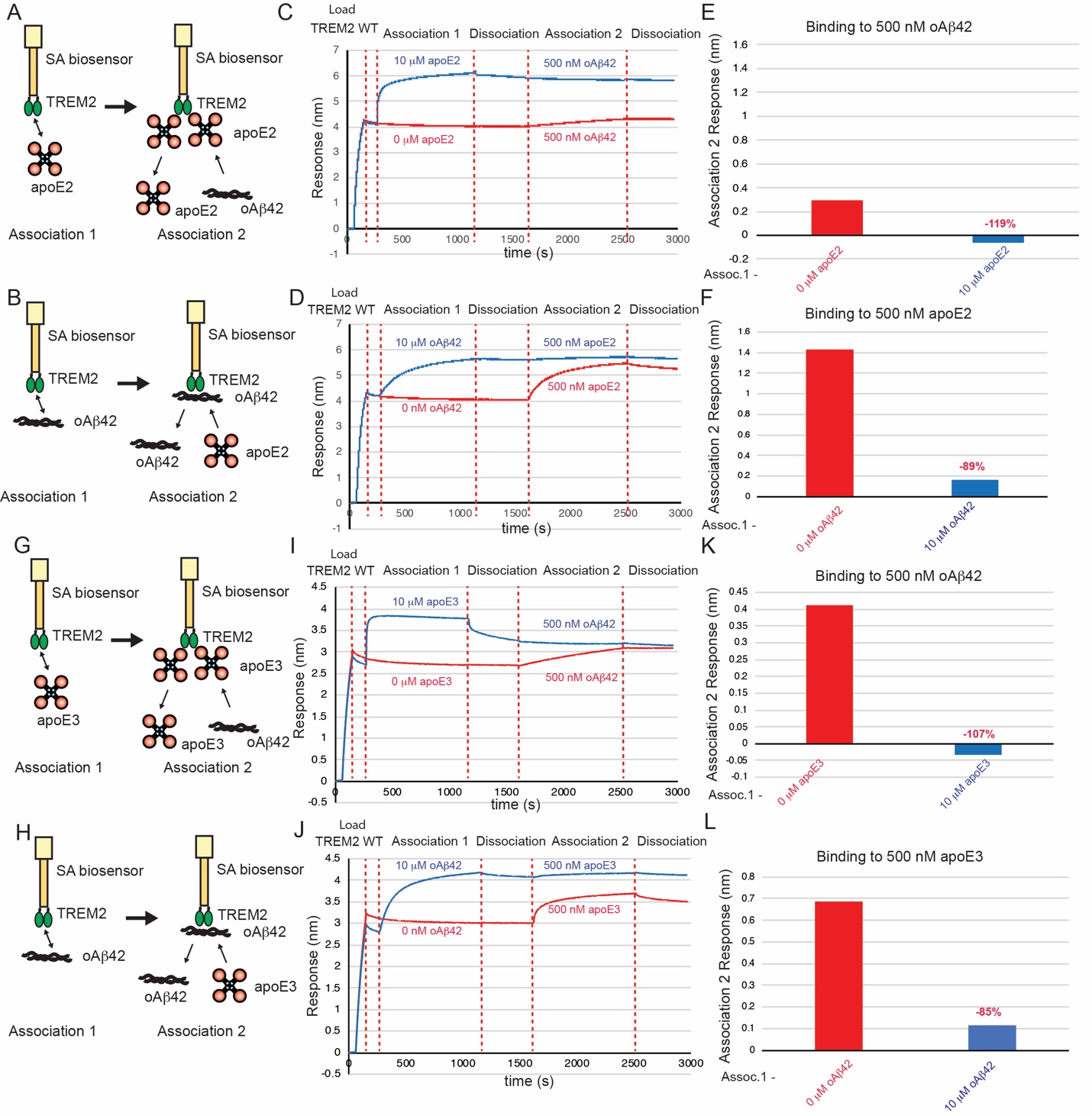


**Supplemental Figure 6**. **ApoE2/3 and oAβ42 compete for binding to TREM2. A&B)** Schematic of competition binding BLI experiments. **C&D)** BLI sensorgrams for **C)** apoE2 competing oAβ42 binding to TREM2 and **D)** oAβ42 competing apoE2 binding to TREM2. Red sensorgrams are TREM2 binding to **C)** 500 nM oAβ42 or **D)** 500 nM apoE2 alone while blue sensorgrams show competition experiments where **C)** 10 μM apoE2 or **D)** 10 μM oAβ42 are bound first. **E&F)** BLI binding magnitudes for TREM2 binding to **E)** 500 nM oAβ42 when pre-binding apoE2 or **F)** 500 nM apoE2 when pre-binding oAβ42. Percent decrease in Association 2 binding signal in the

presence of the competitor is shown above the bars.

**G & H)** Schematic of competition binding BLI experiments. **I&J)** BLI sensorgrams for **I)** apoE3 competing oAβ42 binding to TREM2 and **J)** oAβ42 competing apoE3 binding to TREM2. Red sensorgrams are TREM2 binding to **I)** 500 nM oAβ42 or **J)** 500 nM apoE3 alone while blue sensorgrams show competition experiments where **I)** 10 μM apoE3 or **J)** 10 μM oAβ42 are bound first. **K&L)** BLI binding magnitudes for TREM2 binding to **K)** 500 nM oAβ42 when pre-binding apoE3 or **L)** 500 nM apoE3 when pre-binding oAβ42. Percent decrease in Association 2 binding signal in the presence of the competitor is shown above the bars.


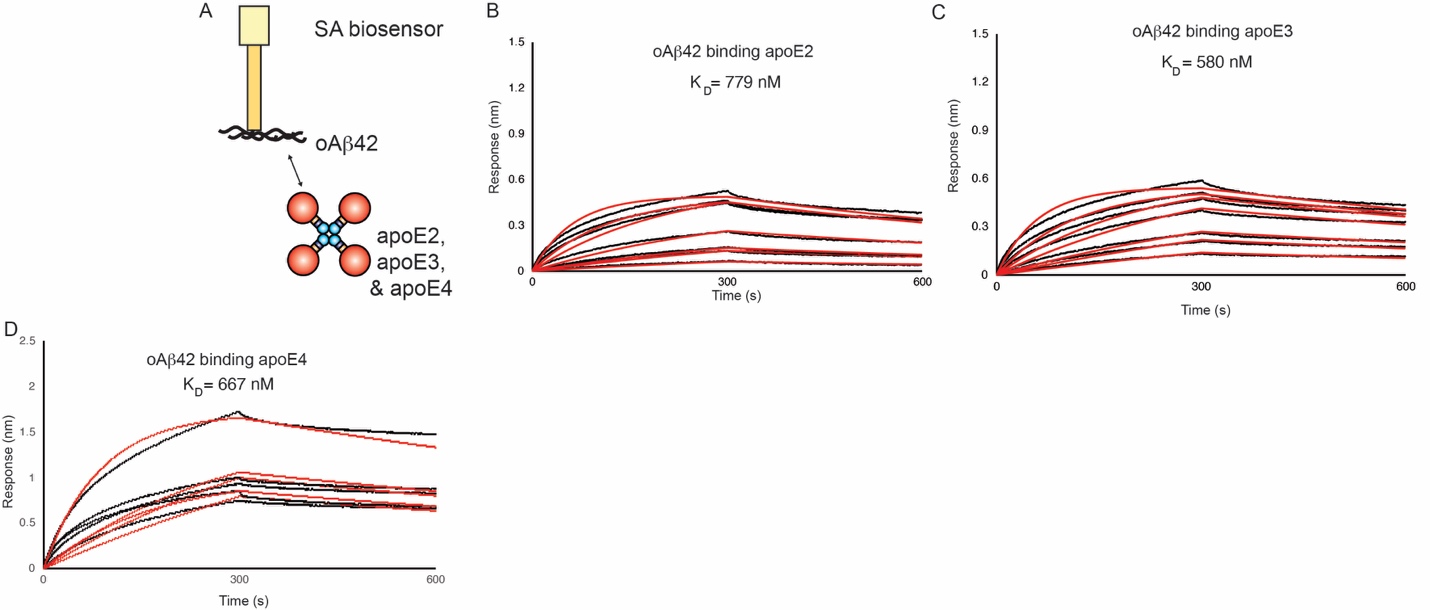


**Supplemental Figure 7**. **oAβ42 binding to apoE2,3,4. A )** Scheme of experiment **B-D)** BLI sensorgrams for oAβ42 binding **B)** apoE2**, C)** apoE3, **D)** apoE4. Black = BLI sensorgrams; red = 1:1 kinetic fits. Derived K_D_s are shown.

**
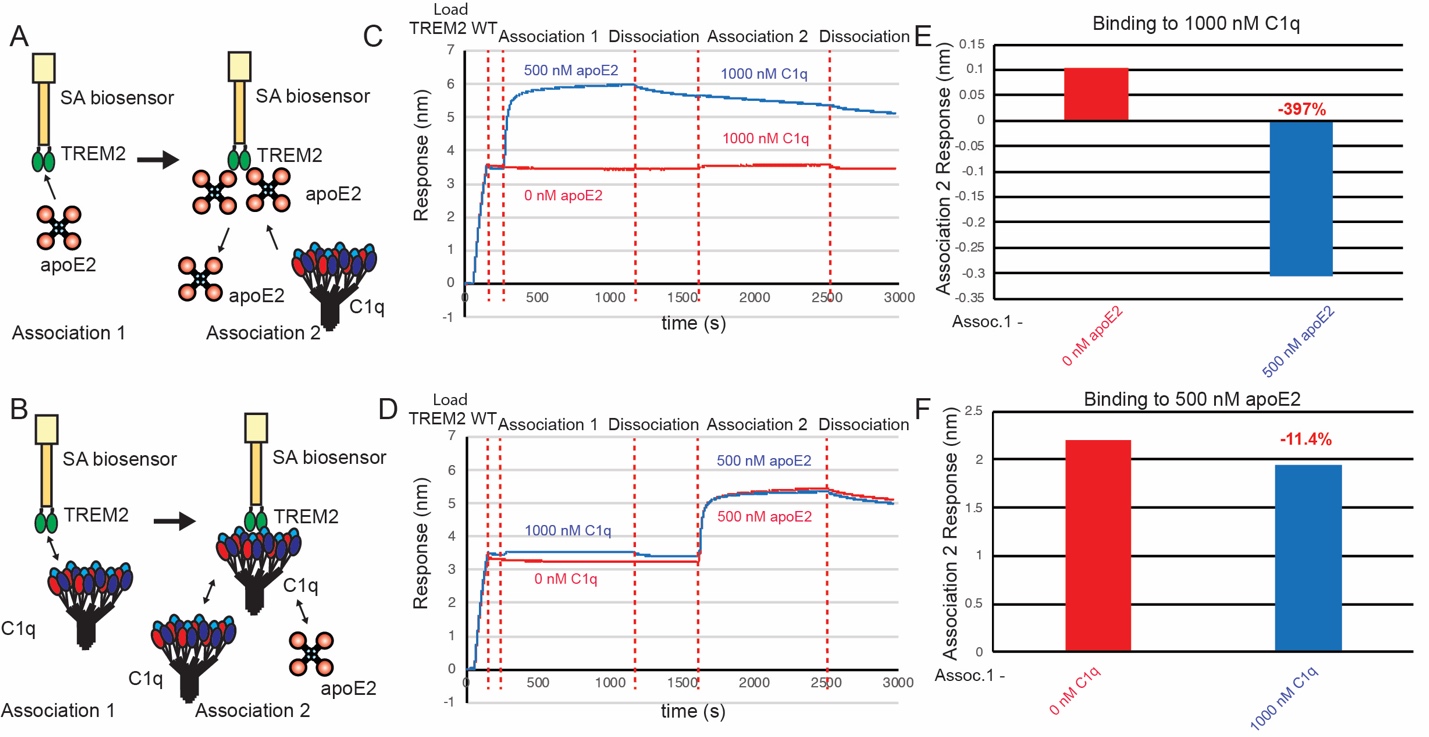
**

**Supplemental Figure 8. C1q minimally competes with apoE2 for binding to TREM2.** **A&B)** Schematic of competition binding BLI experiments. **C&D)** BLI sensorgrams for **C)** apoE2 competing C1q binding to TREM2 and **D)** C1q competing apoE2 binding to TREM2. Red sensorgrams are TREM2 binding to **C)** 1000 nM C1q or **D)** 500 nM apoE2 alone while blue sensorgrams show competition experiments where **C)** 500 nM apoE2 or **D)** 1000 nM C1q are bound first.

**
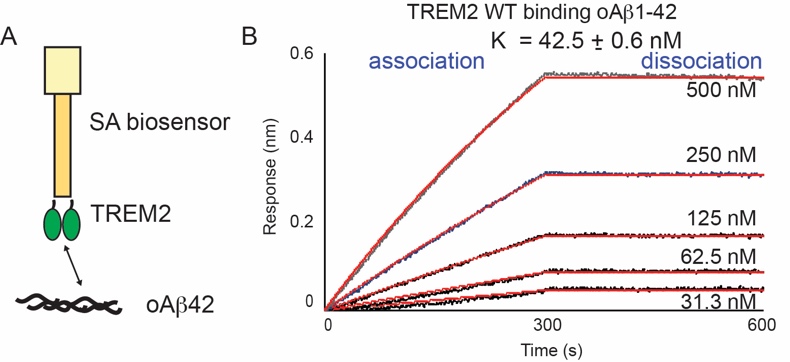
**

**Supplemental Figure 9. TREM2 WT binds to oAb42 with high affinity.** Immobilized TREM2 WT was probed for binding to oligomeric Ab42 (500 – 31.25 nM). **(A)** Scheme of experiment. (**B)** BLI response for TREM2 WT. Double-reference subtracted data (black) overlayed with 1:1 kinetic fits (red). K_D_ was derived from kinetic fits.
